# The social determinants of chronic disease management: perspectives of elderly patients with hypertension from low socio-economic background in Singapore

**DOI:** 10.1186/s12939-018-0897-7

**Published:** 2019-01-03

**Authors:** Sok Teng Tan, Rina Yu Chin Quek, Victoria Haldane, Joel Jun Kai Koh, Emeline Kai Lin Han, Suan Ee Ong, Fiona Leh Hoon Chuah, Helena Legido-Quigley

**Affiliations:** 1 Saw Swee Hock School of Public Health, National University of Singapore and National University Health System, 12 Science Drive 2 #10-01, Tahir Foundation Building, Singapore, 117549 Singapore; 2 London School of Hygiene and Tropical Medicine, WC1H 9SH, London, UK

## Abstract

**Background:**

In Singapore, the burden of hypertension disproportionately falls on the elderly population of low socio-economic status. Despite availability of effective treatment, studies have shown high prevalence of sub-optimal blood pressure control in this group. Poor hypertension management can be attributed to a number of personal factors including awareness, management skills and overall adherence to treatment. However, these factors are also closely linked to a broader range of community and policy factors. This paper explores the perceived social and physical environments of low socio-economic status and elderly patients with hypertension; and how the interplay of factors within these environments influences their ability to mobilise resources for hypertension management.

**Methods:**

In-depth interviews were conducted in English, Chinese, Chinese dialects and Malay with 20 hypertensive patients of various ethnic backgrounds. Purposive sampling was adopted for recruitment of participants from a previous community health screening campaign. Interviews were translated into English and transcribed verbatim. We deductively analysed leveraging on the Social Model of Health to identify key themes, while inductive analysis was used simultaneously to allow sub-themes to emerge.

**Results and discussion:**

Our finding shows that financing is an overarching topic embedded in most themes. Despite the availability of multiple safety nets, some patients were left out and lacked capital to navigate systems effectively, which resulted in delayed treatment or debt. The built environment played a significant role in enabling patients to access care easily and lead a more active lifestyle. A closer look is needed to enhance the capacity of patients with mobility challenges to enjoy equitable access. Furthermore, the establishment of community based elderly centres has enabled patients to engage in meaningful and healthy social activities. In contrast, participants’ descriptions showed that their communication with healthcare professionals remained brief, and that personalised and meaningful interactions that are context and culturally specific are essential to advocate for patients’ overall treatment adherence and lifestyle modification.

**Conclusion:**

Elderly patients with hypertension from lower socio-economic background have various unmet needs in managing their hypertension and other comorbidities. These needs are closely related to broader societal factors such as socio-demographic characteristics, support systems, urban planning and public policies, and health systems factors. Policy decisions to address these needs require an integrated multi-sectoral approach grounded in the principles of health equity.

## Background

Hypertension is the leading independent risk factor for mortality associated with cardiovascular diseases (CVD), chronic kidney disease, and diabetes, which contributed to more than 40% of deaths worldwide in 2010 [1, 2]. It is also one of the most important causes of years lost due to disability and premature mortality [3]. Globally, more than 9 million deaths are attributable to complications of hypertension [4].

Despite availability of effective treatment, prevalence of sub-optimal blood pressure control remains high, leading to the development of CVD in patients [5]. Hypertension can be managed through adherence to medication and lifestyle modifications including physical activity, a low-salt and well-balanced diet, and weight control [6, 7]. However, research has revealed several barriers to patients with hypertension from effectively managing and controlling their blood pressure. These barriers include inaccessibility to home blood pressure monitoring [8], lack of knowledge and awareness of hypertension severity, low self-efficacy hindering uptake of healthier lifestyles, and perceived poor social support [9–13]. Whilst addressing these personal factors is crucial in the efforts of improving population health, policy makers must recognise the significance of “upstream” societal conditions in shaping these “downstream” determinants [14–17].

“Upstream” conditions, including physical and social environments, are widely recognised to underpin health, both in terms of infectious and non-communicable diseases (NCDs) [16–22]. In high-income countries, evidence shows that disease burden is often concentrated among those at the bottom of the social ladder [16, 20, 23–25]. A review conducted by Kaplan and Nunes [26] showed a negative correlation between socioeconomic status (SES), measured by educational status and income, and the incidence of hypertension. Social inequalities affect not only NCD outcomes, but also how patients manage their chronic conditions with the amount of resources they are able to mobilise [15, 27, 28]. Thus, the interplay of societal conditions, individual lifestyles, and biological factors is a crucial consideration in determining policies to improve population health.

Evidence on the association between social inequalities and health disparities has been well established, but little is known about the impact of the physical and social environments on low SES elderly patients with hypertension in Singapore. Thus, we conducted this qualitative study to explore the perceived social and physical environments of low SES, elderly patients with hypertension in Singapore; and how the interplay of different societal factors influences their management of hypertension and their healthcare experiences.

## Case study setting

Situated in the Southeast Asian region with a rapidly ageing population, Singapore is a high-income, multi-ethnic society made up of three main ethnic groups; Chinese (74.3%), Malays (13.4%) and Indians (9.0%) [29]. Health financing in Singapore is deeply ingrained in the principle of individual responsibility which emphasises co-payments to prevent misuses [30]. Alongside, a multi-layered financial protection scheme exists to enhance the population’s access to affordable healthcare. This scheme includes Medisave, a compulsory savings account earmarked for health care expenses and tied to an individual’s income, various national health insurance schemes, and the ultimate safety net, Medifund, a government endowment fund for needy Singaporeans who have exhausted all other means to pay for health services [30]. While there is no official poverty line and no statistics on incidence of poverty in Singapore [31, 32], various financial assistance schemes are in place to assist households with a monthly per capita income of less than S$650 (about US$475) [33]. In 2015, 54% of the 33,759 household main applicants or individuals receiving either short- or long-term financial assistance were 55 years old and above [34]. In addition, healthcare subsidies are provided to low- and middle income households with monthly per capital income of less than S$1800 (about US$1317) through the Community Health Assist Scheme (CHAS) [35]. Additional healthcare subsidies are available for citizens who were born before year 1950 through the Pioneer Generation (PG) package [36].

Despite the decline of prevalence of hypertension among adults aged 30–69 years from 24.9% in 2004 to 23.5% in 2010 [37], hypertension remains a challenge among the elderly in Singapore. According to a study conducted by Malhotra et al. [12], close to 75% of community-dwelling elderly Singaporeans aged 60 and above are diagnosed with hypertension. Among them, 75.9% had sub-optimal blood pressure (BP) control. The Malay ethnic minority group, which constitutes 13.4% of the Singaporean population [29], disproportionately accounted for more than 81% of the elderly hypertension cases, and 88% of those sub-optimal BP control cases [12]. Being a Singapore resident of lower SES was also associated with higher prevalence of hypertension and poorer hypertension management [12]. In recent years, Singapore has introduced several initiatives to address the population’s NCD needs. However, these initiatives lack an integrated multi-sectoral approach to address interactions between “downstream” and “upstream” determinants [38].

## Methods

### Sampling and recruitment

Patients who previously participated in a Neighbourhood Health Screening (NHS) project conducted by the National University of Singapore’s Yong Loo Lin School of Medicine who consented to be contacted for other research purposes were recruited for this qualitative study. Twenty participants who had hypertension, aged 55 years and above, and from the lower SES were identified through the NHS project participant list and recruited through purposive sampling. Lower SES is defined as living in one- or two-bedroom Housing Development Board^1^ (HDB) flats. All participants resided at rental HDB flats at the Central region of Singapore (Kallang, Eunos, and Kampong Glam). Participants were invited to participate in the study and were informed about study objectives and discussion topics via phone calls. Recruitment ceased when all researchers collectively agreed that thematic saturation had been reached, and that data generated from subsequent interviews would be unlikely to lead to new information. This qualitative study was carried out from November 2017 to April 2018. Ethical approval was obtained from the National University of Singapore Institutional Review Board (NUS-IRB). Signed consent forms were obtained from participants, and permission to be audio-recorded and quoted anonymously in research outputs were also granted. To ensure confidentiality, participants were informed that all interview materials would be stored safely and their personal information will not be exposed to anyone outside the research team.

### Data collection

Face-to-face, in-depth interviews were conducted with all 20 participants guided by a semi-structured interview guide. In-depth interview was used to allow participants to discuss their experiences and perceptions in managing their blood pressure through a focussed but free-flowing interaction. Their characteristics are shown in Table 1. Each interview lasted an average of 45 min and was carried out at a place and time of the participants’ preference and convenience. Interviews were conducted in Chinese (Mandarin, Cantonese, or Hokkien), and Malay according to participants’ native languages. All interviews were audio recorded. Field notes were taken during or immediately after the interviews.

**Table 1:** Participants’ demographic characteristics

Patients’ Characteristics	Malay		Chinese		Total
	Male	Female	Male	Female	
Age range					
55–65	1	1	1	2	5
66–75	1	2	1	4	9
75 and above	0	0	2	4	6
Marital Status					
Single	1	0	1	1	3
Married/ Living with partner	2	2	1	4	9
Divorced	0	0	0	1	1
Widowed	1	1	0	3	5
Not mentioned/ unclear	0	0	1	1	2
Living arrangement					
Living alone	2	0	1	2	5
Living with partner only	1	2	0	3	6
Living with partner and/or other family members (children / grandchildren / siblings)	0	1	2	2	5
Living with live-in helpers	0	0	0	2	2
Living with other tenants	0	0	1	1	2
Employment					
Full time	0	0	1	0	1
Part-time	1	0	2	2	5
Retired	1	1	1	3	6
Unable to work due to physical conditions	1	1	0	3	5
Not mentioned/ unclear	0	0	0	3	3

### Data analysis

The interviews were recorded in full and transcribed in verbatim. Professional transcriptionists who were able to transcribe and translate the interviews into English simultaneously were contracted to transcribe the interviews. Transcripts were examined rigorously by the respective interviewers (STT, RYCQ, JJKK, and EKLH) to ensure translation was done literally without losing accuracy. Data generated from the interviews were approached interpretively by focussing on the patients’ experiences, perceptions, and how they made sense of the topics discussed. STT and RYCQ deductively coded the data according to Dahlgren and Whitehead’s Social Model of Health as cited in Dahlgren and Whitehead (2006) [20]. At the same time, the coders also coded line-by-line by assigning code words to every sentence to allow key and sub-themes to emerge. These codes were then grouped into key themes and sub-themes, and eventually organised into broad categories deriving from the Social Model of Health namely i) individual level; ii) community and institutional level; iii) socio-economic status; and iv) systems and policy level. QSR NVivo 11 software was used to manage and organise data. To ensure inter-coder reliability, two coders (STT and RYCQ) coded the data and triangulation was done regularly throughout the analysis process. The two sets of codes were compared and differences were ironed out through discussions until agreement was reached. Unresolved disagreements were sorted out by engaging a third researcher (HLQ) who provided neutral views to the dataset and analysis. The authors (STT, RYCQ, and HLQ) participated in an iterative process of identifying and reviewing emerging themes. Regular group meetings were held to discuss themes and deviant cases, as well as to establish consensus on a final list of themes and key findings within each theme. In this paper, pseudonyms are used and identifying data have been removed to maintain confidentiality. Patients’ pseudonyms and their corresponding basic demographic characteristics are shown in Table 2.

**Table 2:** Participants’ pseudonym and their corresponding characteristics

Pseudonym	Sex	Ethnic
Yeok	Female	Chinese
Gim	Female	Chinese
Chun	Female	Chinese
Eng	Female	Chinese
Hock	Male	Chinese
Kam	Female	Chinese
Ahmad	Male	Malay
Hapsah	Female	Malay
Lian	Female	Chinese
Bee	Female	Chinese
Halimah	Female	Malay
Keng	Female	Chinese
Abdullah	Male	Malay
Hua	Female	Chinese
Meng	Male	Chinese
Kuok	Male	Chinese
Chuan	Male	Chinese
Ling	Female	Chinese
Zaid	Male	Malay
Latifah	Female	Malay

### Reflexivity

STT, RYCQ, JJKK, EKLH, SEO, and FLHC conducted interviews in participants’ language of choice. All interviewers are experienced qualitative researchers trained in conducting individual in-depth interviews. STT and FLHC have professional working experiences with disadvantaged populations and expertise in topics related to social justice and determinants of health. RYCQ is a nutritionist, VH is a researcher in the field of hypertension, and JJKK, EKLH, SEO and HLQ are experienced health policy and systems researchers. All authors have diverse backgrounds and experience; hence, regular discussions throughout the study phase that emphasised on individual reflexivity were essential in ensuring that the interviews and data analysis were approached objectively.

## Results

**Fig. 1:**
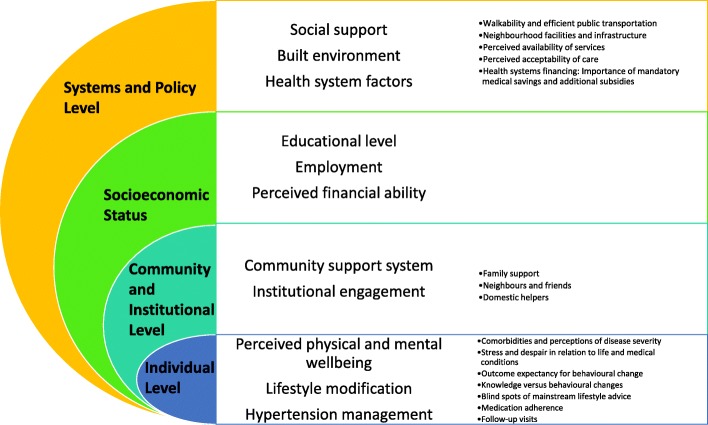
Patients’ perceptions on individual’s conditions, and perceived social and physical environments, adapted from Dahlgren and Whitehead, 1993

Leveraging on Dahlgren and Whitehead’s Social Model of Health, key findings of this study are organised according to Fig. 1. The key findings encompass four interrelated levels – individual, community and institutional levels, SES, and systems and policy level. The innermost layer – the individual level - relates to the participants’ personal experiences and their ability to manage hypertension. The main themes include their perceived physical and mental wellbeing, and aspects related to management of their medical condition. Sub-themes highlighted include participants’ comorbidities and perceptions of the disease severity, stress and despair, behavioural change, and medication taking and follow-up visits.

The next layer represents participants’ social capital, as well as their engagement in relevant activities within the community. This level focused on understanding the various forms of support that participants obtained from family or community and the impact it had on them managing hypertension.

Above the community and social capital level is participants’ SES, which include factors such as education level, employment status, and their perceived financial ability. This level aimed at understanding participants’ background and how these determinants interact with broader factors to underpin patients’ ability to mobilise resources in managing their conditions.

Lastly, the outermost layer of the model represents the broader aspects of society such as the social support system, built environment, and their perceived health system factors that play significant roles in influencing hypertension management. The sub-themes consisted of walkability and efficient public transportation, neighbourhood facilities and infrastructure, perceived availability of services, perceived acceptability of care, and aspects related to health system financing.

### I. INDIVIDUAL LEVEL

#### Perceived physical and mental wellbeing

##### Comorbidities and perceptions of disease severity

Apart from hypertension, most participants reported to have a few other health conditions. The most cited conditions are chronic muscular pain, hyperlipidaemia, heart disease, and diabetes mellitus. A small number of participants also reported to have epilepsy, asthma, or lymphatic obstruction. Most participants expressed their worries over the complications of other diseases such as diabetes or heart disease. On the contrary, they interpreted hypertension as a common illness that can be easily controlled. Gim described *“Hypertension, I am not scared… A lot of people have hypertension, I’m not too worried about it. [Among] 10 people, 9 people have high blood pressure.”*

In contrast, a number of participants viewed that all chronic conditions, including hypertension, require equal attention and acknowledged the need to control their conditions adequately. Several participants stated their fear of the consequences of uncontrolled hypertension that could impact their lives significantly.

##### Stress and despair in relation to life and medical conditions

Many participants expressed distress when talked about their lives and medical conditions. A few participants who had conditions such as prolonged cough or chronic pain emphasised that their priority was to treat these conditions that are of immediate troubles in their lives. These conditions were also often the source of stress and helplessness, as highlighted by Latifah “*The one that influences me is that I can’t control, I can’t fight, I can’t bear is the [weakened] leg and stomach [pain].”* The distress caused by various medical conditions also led some participants to avoid discussing any of them. The denial and despair were apparent when support was perceived to be inadequate, as illustrated by Kuok who had withdrawn from participating in any social activities, *“It’s very painful to be sick. I don’t think about anything. I won’t try to think about anything. […] Sometimes, I’m sick and it hurts here and there. I’d wonder why other people leave this earth so fast but I am always like this. I’m neither dead nor alive. It’s very tough and headache-inducing.”* A number of other participants also shared their worry, anxiety, and grief over other life events and perceived that these feelings have contributed to high blood pressure; hence, a way to manage their blood pressure is to have their feelings in control. Hapsah when discussed about the cause of her hypertension highlighted, *“I have so many problems to think about... How am I not getting sick? Huh? Surely get sick right? I get worried thinking about my late son... and all these… Okay… But I’m the one who will be dead… I’m sleepless you know...”* While their physical conditions are often addressed promptly, none of the participants mentioned the intention to seek help for their mental health.

#### Lifestyle modification

##### Outcome expectancy for behavioural change

Participants had mixed responses on the reasons they change or maintain their lifestyle. The main reasons to change were tied to expected outcomes such as avoiding disease progression, the feeling of being in control, avoiding hefty medical bills in the future for management of complications, and positive effects of physical activity. Yeok explained, “*If you do not protect yourself, who would protect? Others cannot protect you… If [you have] high blood pressure and do not control yourself, if [you] collapse… heart does not die, body dies… So you just lay there and it’s awful… and become a burden to others.”*

There were several unique responses under this theme. One participant with lower health literacy and reported previous financial difficulty in accessing hypertensive treatment resorted to using physical activity to compensate for medication non-adherence. The only participant who smoked reported that she tried to reduce smoking. However, she cited cost as a main reason for quitting and not health.

##### Knowledge versus behavioural changes

Participants claimed that they received information from healthcare professionals and various other sources on dietary requirements to manage their hypertensive condition. Some emphasised the importance of self-control in maintaining health, as illustrated by Chun, *“I see doctor, the doctor told me. I also got to know from the television. To keep healthy is not about relying on others, it’s about relying on yourself and your own will power.”* While most of them endeavour to strictly follow the advice provided, dietary change was more challenging for other participants. The majority of participants informed that they usually cook at home and are therefore able to intentionally reduce salt in their food or opt for healthier options. However, a few indicated that they occasionally take a diet break to satisfy cravings. A number of the participants stated that they usually eat-out or take-away thus are unable to control salt content in their food. Nevertheless, during counter-probing, a handful of participants gave conflicted accounts of reducing salt intake but in practice they prepare food of high salt content such as pickle, or high fat content such as curry or pork belly.

##### Blind spots of mainstream lifestyle advice: Cultural perspectives, physical condition, and other commitments

Participants’ responses indicated that lifestyle advice given by doctors and nurses lack perspectives that cater to individual’s contexts and needs. A small number of participants also showed frustrations because dietary advice given by healthcare providers was not tailored to their food culture, as complained by Hapsah, “*They advise taking this like... Oat, green veggies that Chinese like to take as soup, steamed fish... We Malays can’t eat like that... You know Malays must eat with chilies, without chillies we can’t eat right?”* On the other hand, almost all participants indicated that they perform at least light physical activities such as walking or participating in elderly group workout sessions organised by senior activity centres. A handful of participants emphasised that their physical condition or immobility has deterred them from carrying out any physical activity. Abdullah highlighted, “*Because I cannot hike, I’m afraid of my heart. Ah... I cannot run.”* Other participants also highlighted their work and family commitments have hindered them from adequately exercising, as illustrated by Keng, *“Yes, before my grandchildren came along I did Yoga and swam, but after my grandchildren were born I gave up on exercise to take care of them.”*

#### Hypertension management

##### Medication adherence

Most participants accounted that they strictly adhered to taking hypertensive medication with assistance from various forms of reminders such as weekly pill organisers, placing medication in obvious places, and linking medication to mealtimes. Eng described, *“Because I keep my medication here… This bag. When I get up in the morning, I put it on the table. After I eat food, I take medication.”* Among reasons given by participants to adhere to their medication include wanting to take control of their own body and unbearable symptoms. Despite the motivation, a few participants were frank about missing pills at times but stressed that they usually endeavour to take them as soon as they remembered.

A small number of participants manifested their difficulties in adhering to medication due to multiple factors such as lacking means to access treatment, side effects and feeling frustrated with taking medications, as depicted by Meng, “*I don’t want to. I feel very tired from taking the medication.”*

##### Follow-up visits

All except two participants informed that they went for follow-up visits every 2 to 6 months, depending on the severity of their condition. Most participants received treatment at polyclinics, a small number reported accessing private services to avoid long waiting times and transportation issues. Two Malay participants admitted to not complying with follow-up visits. In one case, it was due to refusal of services at private clinics as the funds in her Medisave account had depleted. In the second case, the participant was avoiding getting medications due to side effects.

### II. COMMUNITY AND INSTITUTIONAL LEVEL

#### Community support system

Participants’ accounts revealed a complex network of community support which enables elderly with chronic conditions to receive various forms of allowance, assistance, provision, as well as emotional support. These forms of support can be further divided into the following sub-themes:

##### Family support

Most participants reported to have maintained close contact with, and received financial support from their children or grandchildren, and some sought comfort from their partners’ companionship. Eng described when asked about her family, “*Somebody [children] comes every week. […] I take care of myself. So I rent out a room… So they… my sons, some give me 200 a month, 100 a month… [laugh] I don’t want them to give more.”* Despite regular contact with their children, these participants related that they are independent in managing their medical conditions; and perceived that children can offer no substantial help in alleviating their medical problems. A few participants highlighted their frustration related to their children’s neglect and emphasised that they were empowered to live independently. Gim complained, *“My son refuses to care for me, so I have to care for myself. I reported to the Social Services Offices, 450 a month, how is it enough?”*

##### Neighbours and friends

Some participants reiterated the importance of building network with neighbours and friends within their residential areas for immediate emotional support as well as timely assistance in the event of an emergency. Hapsah emphasised, “*We stay here in this old folk’s home we must make friends if we don’t then later if got something for help will be difficult right?”* This community bonding facilitates discussion among elderly about their health issues and insights. Some participants also shared that they look out for needy neighbours and assist them, as narrated by Gim, *“[…] I take her [neighbour] to [polyclinic]. Previously, she was a lot worse, [she] had to use a wheelchair, I take care of her all along. […] We do grocery shopping together and she buys this and that; and I will tell her, ‘Aunty, don’t buy too much of this, it’s very oily; if you want, this or that is better, it has no fat [and] no oil.’”*

##### Domestic helpers

Two participants were supported by domestic helpers in their daily chores and management of medical conditions. They explained that the assistance obtained from domestic helpers had effectively addressed their immediate needs for daily routine and treatment adherence, as related by Hua, “*[My helper] organises the medications into boxes for me. […] She definitely goes with me [to see a doctor]. […] Doctors teach her, she would prepare them for me.”*

#### Institutional engagement

Most participants who are not working demonstrated keen participation in activities organised by community centres or senior care organisations at their residential areas. They complimented that these organisations offer great opportunities for elderly to engage in community activities. A number of participants also illustrated their religious affiliations which either brought comfort in times of despair or enabled them to be socially active by being involved in religious events. Latifah expressed, “*[…] because I have fallen many times already before so if I fall again then nothing that they can do... maybe I’ll be bedridden... that’s why I’m really careful... control and stay strong... seek help from ALLAH.”*

Nevertheless, a small number of participants shared that they seldom or never participated in any community activities; most did not share a reason, but one cited physical immobility as a barrier.

### III. SOCIAL ECONOMIC STATUS

#### Education level

A number of participants indicated that they either received no education or had only a few years of primary education. Despite that, some of them highlighted they picked up some reading and speaking skills on their own. Others who received some education reported to have used their literacy skills to obtain health information or assist themselves in treatment adherence.

#### Employment

As Medisave is tied to individual income, participants’ former and current employments play a major role in determining the amount of medical savings they can mobilise to address their health problems. Many participants reported to have retired. Most of them had previously been employed as manual labourers such as cleaners, market vendors, construction workers, panel beaters, security guards, and storekeepers. A few participants shared that they had regular jobs and consistent income, while others had odd jobs with irregular income. Due to limited funds in their Medisave, a number of participants with comorbidities indicated that their medical saving accounts had depleted. Some working participants claimed that they had to change jobs according to their physical ability. They reported to continue working despite having exceeded retirement age, demonstrating high resilience when perceived access to financial support is difficult. Ling shared her reason of continuing two cleaning jobs a day, “*I work part-time two hours in the morning, four hours in the afternoon. […] Washing toilets […] I’m now holding two jobs and it’s going well so I might as well keep at it until I can no longer work and I stop and don’t work anymore. Right? Better not stop and save up some money first.”*

#### Perceived financial ability

All participants were of lower SES and confirmed that they had to pay for daily necessities such as house rent, utility bills, and food. A few were able to access full public assistance, which provided them with heavily subsidised public housing, monthly allowances, and free healthcare at public healthcare facilities. A small number iterated that the assistance amount is insufficient for their daily expenses. Several participants reported to be living on the margins and needing to strategically manage their finances to make ends meet. Many also highlighted having uncertain or irregular financial support.

### IV. SYSTEMS AND POLICY LEVEL

#### Social support

Participants reported a number of social support mechanisms. Almost half of the participants indicated to have received financial support in various forms from the Social Service Offices.^2^ A few participants mentioned that they regularly received food parcels distributed by some charity organisations. Most ethnic Malay participants highlighted that support from their children is minimal and mostly irregular. This prompted them to approach other sources of support but were often left in a situation of uncertainty as support was usually short-term. A few Malay participants also complained that they did not know how to navigate the social support system despite having dire needs. Halimah expressed her frustration, “*So I did a request for help... [I] gave them the payslip this and that but still under process. […] then they replied me with a letter in green telling that my application was rejected, so what can I do?”*

#### Built environment

##### Walkability and efficient public transportation

A walkable environment as well as efficient and affordable public transportation eased most participants’ physical access to health facilities. The majority of the participants reported commuting to the polyclinics via public buses. Most of them explained that they are independent and did not require a caregiver to go with them, as depicted below:

*Kam: Now I can take a bus, bring a crutch and take a bus. Take two buses, 100 to Geylang, alight, then 80.*

*Interviewer: Is it difficult to travel like this?*

*Kam: No.*

*Interviewer: Do you need to walk a long distance?*

*Kam: No. I take a break when my legs sore, break for a while then walk again.*

However, a few participants perceived that due to their immobility, they were unable to take public transport and had to rely only on taxi to commute to and from clinic visits or opted for expensive private mobile services that are delivered to residential areas. These participants also complained about the high out-of-pocket payment for transportation to access care. Zaid shared, *“St Andrew [is] just downstairs only. I can take my wheel and go [laugh] I don’t need to take a taxi, because how is five hundred dollars [a month] enough [if I] want to go here and there? Unless they [government] can give me claim for the taxi fare right?”*

##### Neighbourhood facilities and infrastructure

Most participants reported including exercise in their daily activities and this was made possible with the implementation of facilities such as the Peace Connect – St Andrew’s Seniors’ Activity Centre where physical rehabilitation was available, and the outdoor gym equipment were established within the neighbourhood. Some participants added that they would go downstairs for walks with their friends in their free time. As Hua explained:

*Interviewer: Auntie, do you go downstairs to walk?*

*Hua: Yes. There are the swinging ones [gym equipment], they have everything… It’s downstairs, opposite...*

*Interviewer: So do you have friends that do it with you?*

*Hua: Yes. Quite many people do exercise here.*

#### Health system factors

**Perceived availability of services**

##### Location and accessibility

Participants’ narrations exhibited that private health facilities such as Traditional Chinese Medicine (TCM) providers and General Practitioner (GP) services are abundant and within the vicinity of their residential areas. For minor illnesses that require immediate attention, some participants denoted their preference for these neighbourhood facilities over polyclinics that are usually located at a distance away from their residence. Despite the distance, most participants specified that they received treatment for chronic conditions from polyclinics and this was enabled by a friendly built environment.

##### Waiting time

A few participants reported that the waiting time at polyclinics has improved tremendously compared to in the past. They attributed the improvement in waiting time to the implementation of the “appointment system” where they were provided with appointment date and time slots.

Nevertheless, many participants complained about the long waiting time at the clinics despite having prior scheduled appointments. While none of the participants reported defaulting treatment due to waiting time, most participants expressed that they had no other options but to withstand long wait for more affordable services. Those who had the means to reported resorting to seeking private services to avoid frustration, such as Bee, who shared, *“I visit a private clinic now, not the government facilities. […] The queue [at the government facilities] is too long and a lot of people.”* There were also a handful of participants who understood that it was inevitable for the long wait as they were not the only patients the doctor had.

**Perceived acceptability of care**

##### Communication with healthcare professionals

Participants depicted communication with healthcare professionals as brief, healthcare information provided was usually generic, and the main focus was on their immediate health status. The response below from Gim was typical:

*“They ask me how I am of late, whether I am better, then I say ‘never get better one la at most just take your medicine to prevent sickness’. He will say ‘need to be careful and don’t eat food that is too oily, and don’t be too angry too much. Being too angry, your blood pressure will shoot up.’ I said I know. Like that only.”*

##### Language

Most participants reported that they were satisfied and contented with the services provided by healthcare professionals. They related that if they encountered doctors who were unable to speak their language, a translator would be called in to assist. Just as Halimah explained during the interview: “*[...] if I don’t understand, he will call for Malay to explain to me [...] I would prefer Malay because we understand what they say...”* However, Kuok manifested his struggle to communicate effectively with foreign healthcare professionals, *“You know the doctors in the clinic nowadays are all foreigners. Even if you tell them, they don’t really understand what you are talking about and they won’t accept you. There were many doctors from China and Vietnam. Sometimes when you talk to them, it’s like a chicken talking to a duck.”*

##### Healthcare professionals’ attitudes

Most participants related that healthcare professionals nowadays have a lot more patience and empathy unlike those in the past where they would raise their voice at them for no apparent reason. Some further expressed that they are satisfied with the care received and that no additional improvement was required. Yeok joyfully recounted her experience with the healthcare professionals: “*The nurses now are very nice, they don’t scold anyone. [They] know everything. You ask them, they explain to you, talk to you [...] In the olden times […] If you asked them, they would scold you […] If you don’t know [where to] walk… where or which one, they would tell you which room […] they would bring you [there].”*

A few other participants commented that doctors and nurses are impatient, insincere, and disengaged when they communicate with patients. Some of these participants emphasised that healthcare professionals do not take time to understand patients’ condition and explain their medical status, as evident by Latifah, *“[…] but if you ask me to walk and exercise... I’m alone, I’m using a walking stick... What if I fall? How? He doesn’t understand... then when I go and ask for the medicine that the last time he gave one which it helps me reduce the pain right [showing action of typing on a keyboard] Okay... he doesn’t even look at our face or check... nothing [showing action of typing on a keyboard]... computer.”*

##### Perceived disagreement and flawed experiences lead to mistrust

Although most participants displayed high regards for healthcare professionals, many did not feel comfortable about sharing their views with doctors, especially when these views are perceived to be contradictory to doctors’ advices. A few participants shared that they were not willing to inform doctors about taking TCM or other supplements. These participants shared that they felt it is unnecessary to inform doctors about it or they were afraid they would be reprimanded by the doctors and doctors would have the impression that their medical condition is under control. When Kam was asked whether she informed doctor about her replacing hypercholesterolemia medicines with a supplement, she exclaimed, *“No, I don’t dare to tell, in that case I would get scolded.”*

Having gone through flawed experiences with healthcare professionals, a number of participants explicitly questioned doctors’ professional skills and judgement, and some were of the view that doctors are textbook-oriented without considering the reality and context. Chuan illustrated his disappointment in doctors: “*[...] To be honest, the doctors are using us as experimental subjects. I visited a private doctor because my skin has spots and it worsened after I applied the cream. The next day, the doctor changed the prescription. It didn’t work so we changed to another one. It didn’t work either. I asked him how many changes will there be for it to work. It became worse with the applications of cream.*”

**Health systems financing: Importance of mandatory medical savings and additional subsidies**

##### Expensive healthcare covered for by financial protection measures

Overall, participants emphasised that healthcare is expensive in Singapore; however, with existing financial protection measures, most participants found final medical bills affordable, as emphasised by Gim, “[…] *if no help [medical subsidies] I am in trouble I go where and find money! If I got no help, I might as well die and get it done. If short of the pioneer generation [subsidy], I said might as well die and get it done. No one gives me money.”* Almost all participants had Medisave and reported to use Medisave as their first line of defence. Some participants highlighted that after the expansion of Medisave coverage, they pay minimal out-of-pocket (OOP) payment for hypertensive medications. This minimal OOP payment is also further offset by the availability of PG subsidies for some participants. Most participants perceived Medisave as an insurance or a subsidy from the government, hence they did not feel that the medical fees deducted were from their own savings. However, a few participants emphasised that when the yearly ceilings of Medisave and other subsidies are exceeded, the medical fees or co-payments usually takes a toll on their financial situation and they are only able to afford them if they have income. Hock mentioned “*I don’t have to pay cash. There’s a limit; I have to pay cash if the Medisave account saving is used up at the end of the year. […] If I don’t have salary, it’d be quite heavy. Like now, I have a half-day work so it’s still manageable.”*

##### Lack of financial means leading to debt or delayed seeking treatment

Most participants of Chinese ethnic origin reported sufficient Medisave funds to pay for treatment and noted that they found co-payments affordable. Conversely, most Malay participants iterated their difficulty in accessing assistance and paying medical bills. These Malay participants highlighted their struggles in paying for healthcare due to depleted funds in MediSave, having co-morbidities that are not included for heavy subsidies or due to opportunity costs like transportation. Hapsah recounted, “*I do take the medicines, but all medicines are expensive […] how can I survive? I’m not working, they take from half Medisave […] But my Medisave not much left […] so when they deduct about 50 dollars then the remaining two hundred over who has to pay? We have to pay cash but I don’t have money... and sometimes I owe them.”*

Most participants claimed that medical bills related to hypertension are largely covered or offset by existing safety nets offered by the government. However, a few participants highlighted their difficulty in obtaining appropriate care for other illnesses such as chronic pain and certain infections due to a lack of financial means, as demonstrated by Halimah who was experiencing prolonged cough, “*[…] [L]ater I go when I have some money. Now to go there also need some money right? [coughing] My son said when he gets his pay then he will send me to the doctor [coughing].”*

## Discussion

This study provides an in-depth understanding of the perspectives of elderly hypertensive patients of lower SES in Singapore on managing their condition and their healthcare experiences. Our analysis demonstrated that patients’ knowledge and skills in managing their conditions are underpinned by their education background, and interactions with broader community engagement and SES. This is aligned with existing evidence in the literature: a community-based case-control study in Singapore also reported that SES is strongly associated with the awareness, treatment and control of patients’ hypertensive condition. It showed that being of lower SES was negatively associated with hypertension management compared to being of higher SES [39]. Furthermore, our participants highlighted that communication with health professionals was unsatisfactory in empowering them to adopt a healthy lifestyle. This finding is supported by a another study conducted in Asia, which found a gap in beliefs and expectation among healthcare professionals and patients in hypertension management, and suggested that personalised explanation on need-specific lifestyle modification delivered by healthcare professionals could be advantageous [40]. This accentuates the need for a closer look into behavioural change interventions as well as healthcare professional’s’ skills in prescribing lifestyle modification for hypertension management for elderly patients in Singapore.

Most participants in this study perceived hypertension as a disease of less impact to their lives compared to diabetes. This finding echoes studies from other settings, where hypertensive patients expressed the views that other diseases such as diabetes are more important than hypertension [41, 42]. The perceived less severity also led to some patients’ non-adherence to hypertension treatment [42]. This ascertains hypertension’s position of “silent killer” and needs scrutiny. In view of the low hypertension awareness, and treatment and control rate, Canada rigorously implemented extensive hypertension management programmes that have successfully increased hypertension treatment and control rate from 13 to 66% within 20 years [43]. While mirroring such programmes may not be feasible considering differences in contexts, Singapore may benefit from referring to the lessons learned in the policy making process.

Community engagement, built environment, socio-cultural context, and health system factors such as communication with healthcare professionals were among the topics raised when patients discussed how they managed their hypertension. A meta-analysis on influences of social support in hypertension treatment adherence indicated that functional social support significantly and positively contributed to overall treatment adherence [44]. Similarly, our analysis showed that senior citizen activity centres in elderly concentrated residential areas catalysed behavioural changes and increased awareness amongst some participants. A qualitative study in Singapore suggested that elderly persons’ social engagement is influenced by health, financial security and other psychological reasons [45]. Our analysis showed that participants’ mental health condition is often inadequately addressed. This prompted the need to further enhance social policies in order to effectively address elderly’s mental health issues and engage them in beneficial community activities. On the other hand, our analysis showed that neighbourhood walkability and public exercise facilities have enabled patients to lead more active lifestyles. This finding corresponds with a large cohort study in the United Kingdom which concluded that neighbourhood walkability is a protective factor of blood pressure outcomes [46]. However, such convenience is not appreciated by patients with immobility; implying the needs to improve patients’ self-efficacy in self-management, and for a more inclusive approach in designing built environment.

Participants’ accounts also suggested that health system factors such as communication with healthcare professionals played a crucial role in facilitating hypertension management. Study from other South East Asia setting shows that paternalistic consultation style is predominantly used in doctor-patient communication, regardless of patients’ education background. The same study also highlights that consultation contents are mostly medical oriented with little attention for participants’ socio-emotional conditions [47]. Effective communication between healthcare professionals and patients, and meaningful patient engagement in deciding treatment strategies are proven to be positively associated with diabetes self-management [48, 49]. Hence, policies targeting at patient-centred care, patient engagement, strengthening healthcare professionals’ communication skills, and patient empowerment may be beneficial in improving overall NCD management.

Patients’ financial abilities are made complicated by the interactions between their SES and relevant public policies. Studies in many countries showed that health system financing is a significant determinant in improving hypertension control, treatment adherence and outcome [50]. In theory, Singapore has a comprehensive multi-layer financial protection system. However, a more targeted approach is essential to effectively reach the marginalised and vulnerable groups. Patients in this study are of low SES, most were manual labourers with low income, and essentially had limited funds available in their Medisave accounts to afford treatments. With the surge of NCDs among the low SES population groups, the equitability, feasibility and sustainability of the current arrangement are in an urgent need for review. Besides, the process of applying for social support and financial assistance should be made simpler and friendlier to vulnerable groups. This system has been criticised for its intrusive means testing [51] which intuitively deters the most vulnerable from effectively accessing it. In organising healthcare financing, the associated opportunity costs incurred from accessing health services should also be part of the fundamental consideration [52] to ensure equity in access to essential care across groups of various abilities.

Our analysis also shows that the Chinese participants fared better than the Malay participants in managing hypertension. This echoes a cross-sectional study in Singapore where the Malay subgroup was found to have disproportionate high prevalence of sub-optimal blood pressure control [12]. Health inequality and inequity across ethnic groups is not unique to Singapore. Similarly, minority women in the United States are reported to have lower rates of awareness and high rates of CVD mortality and risk factors [53]; and blood pressure control is generally poorer among ethnic minority groups in the Netherlands [54]. Future research should consider exploring disparities and different realities across ethnic groups in Singapore to inform policy decision relevant to ethnic health disparities.

Overall, patients’ enabling and disabling environments in managing their conditions are summarised in Table 3.

**Table 3:** Patients’ enabling and disabling environments in managing their hypertensive condition

Personal factors	Enabling environment	Disabling environment
Knowledge and Skills	• Good literacy contributes to better understanding. • Positive community engagement at residential areas enhances patients’ exposure and opportunities to gain knowledge and skills in managing chronic conditions. • Health information on media (Television and radio programmes, social media, and the Internet) facilitate patients’ understanding of chronic conditions.	• Generic and impersonalised health messages provided by healthcare professionals are ineffective in creating awareness. • Illiteracy or low education level may compromise patients’ understanding of their conditions.
Management of hypertension (Lifestyle, medications, follow-ups)	• Availability of pedestrian pathway and outdoor gym at residential areas facilitate patients’ active lifestyle. • Physical activity sessions organised by NGOs for elderly at residential areas assist patients to lead active lifestyle. • Efficient public transportation system allows physically-abled patients to access care at low costs. • Good availability of polyclinics and appointment system enable patients to access care easily.	• Wheelchair-bound patients struggle to use low-cost public transports, resort to taking more expensive taxis or receiving more expensive mobile services delivered by private practitioners. • Healthcare professionals are not effective in communicating lifestyle information to empower changes. • Mistrust between some patients and healthcare professionals compromises communication, potentially leading to undetected non-adherence of medication • Side effects deter some patients from taking medications. • Some patients lack financial means to adhere to follow-up schedule.
Financial ability to afford care	• Availability of multi-layer financial protection measure eases most patients’ burden in affording care. • Additional subsidies for chronic conditions and for eligible patients enable participants to mobilise their resources more effectively. • Being able to obtain employment post-retirement has provided some patients with income to afford medical care.	• Costs associated with seeking care, especially for immobile patients, are hindering access to health facilities. • Lack of knowledge or means to navigate available assistance contributes to compromised ability to afford care. • Low income results in low MediSave funds which essentially depletes quickly when patients have co-morbidities.

## Strengths and limitations

To our knowledge, this is the first qualitative study in Singapore to explore low SES and elderly hypertensive patients’ social and physical environment. The insights will provide practical implications for informing future policies targeting at improving the overall health of the low SES and elderly populations in Singapore. In addition, patients from different ethnic and dialect groups were interviewed to allow a breadth of views and perspectives, these views were compared, analysed and presented to fill in the existing knowledge gap in the field. Another strength of this study is in the data collection method where in-depth interviews were used to allow topics beyond the interview guide to emerge, which fundamentally contributes to a more complete understanding of how patients perceive their reality.

However, our sampling strategy may have limited our reach to patients who are marginalised. This sampling bias could potentially constrain our analysis to only the “more general” population without including those who are most in need. Further, our study may be limited by desirability bias as our patients generally presented positive interpretations of their experiences, despite some complaints about healthcare professional’s disengagement; similarly evidence has shown elderly patients are more likely to report greater satisfaction in health care services than younger groups [55]. Finally, as the focus of the study was limited to patients’ experiences in terms of hypertension management, we could not do an in-depth exploration of how ethnic differences impact all aspects of patient access to and acceptance of health care in Singapore. This should be a focus of future research on patient experiences in Singapore.

## Conclusion

The ability of low SES, and elderly patients with hypertension to manage hypertension and its’ outcomes are underpinned by a wide range of individual, community, systemic and policy factors. In the endeavour to provide universal health coverage and achieve health equity, it will be beneficial for Singapore to take a closer look into the equitability of existing health financing policies. All in all, addressing the root causes that impede attainment of good health should go beyond health sector’s jurisdiction, and require an integrated multi-sectoral approach.

## Abbreviations

BP: Blood pressure
CHAS: Community Health Assistance Scheme
CVD: Cardiovascular diseases
NCDs: Non-communicable diseases
NHS: Neighbourhood Health Screening
OOP: Out-of-pocket
PG: Pioneer Generation
SES: Socioeconomic status

## Footnotes

1 Singapore’s authority that builds affordable public housing. Households with monthly income of less than S$1500 (about USD1,100) are eligible to rent HDB flats under the Public Rental Scheme.

2 Social Services Offices were set up by the Ministry of Social and Family Development at various HDB towns to coordinate and administer assistance for the public.
